# A rare case of adenocarcinoma of an ectopic pancreas: A case report

**DOI:** 10.1016/j.ijscr.2022.107061

**Published:** 2022-04-08

**Authors:** Kalaji Manhal, Molle Gaetan, Delaunoit Thierry, Mansvelt Baudouin

**Affiliations:** a Digestive Surgery Unit, CHC Jolimont Hospital, la Louvière, Belgium; b Oncology Unit, CHC Jolimont Hospital, la Louvière, Belgium

**Keywords:** CEA, Carcinoembryonic antigen, CA 19.9, Carbohydrate antigen 19–9, ED, Emergencies Department, GIST, Gastro Intestinal Stromal Tumor, EP, Ectopic Pancreas, CRP, C-reactive protein, PPI, Proton Pump Inhibitors, Ectopic pancreas, Adenocarcinoma, Stomach

## Abstract

**Introduction:**

Ectopic pancreas refers to the presence of pancreatic tissue in an unusual anatomical location that has neither anatomic nor vascular continuity with the pancreas. Adenocarcinoma of an ectopic pancreas is rare; only few cases have been described in the literature.

**Presentation of case:**

We reported a 69-year-old man who was admitted to the emergency department with feculent vomiting lasting two days prior to presentation. Endoscopy revealed pyloric stenosis. Biopsies performed during gastroscopy were in keeping with duodenal cancer, while those performed during endoscopic ultrasound suggested a gastric tumor.

A subtotal gastrectomy was done because of results of the extemporaneous analysis and the suspicion of a gastric cancer. The final histopathological report revealed adenocarcinoma of an ectopic pancreas.

**Discussion:**

Malignant transformation of an ectopic pancreas is very rare, and often occurs as an adenocarcinoma. A differential diagnosis of a gastric adenocarcinoma and a gastrointestinal stromal tumor is essential before treatment.

Outcome and a literature review of the pathology, prognosis, and treatment will be discussed.

**Conclusion:**

Despite adenocarcinoma of an ectopic pancreas being rare, it should be considered as a differential diagnosis of submucosal tumors. The standard treatment for ectopic pancreatic adenocarcinoma is surgery, with a higher survival rate of 5 years compared to pancreatic cancer. Frozen section analysis is essential because it will give an idea on the origin of the neoplasia and allow adaptation of the surgical procedure.

## Highlights

• This case report describes a case of adenocarcinoma of ectopic pancreas.
• Malignant transformation of the aberrant pancreas is very rare.
• Partial gastrectomy and duodenectomy of D1 was performed and sample sent for extemporaneous analysis.
• The standard treatment for ectopic pancreatic adenocarcinoma is surgery.

This work has been reported in line with the SCARE 2020 criteria [1].

## 1 Introduction

An ectopic or aberrant pancreas refers to the presence of pancreatic tissue in an unusual anatomical location that has neither anatomic nor vascular continuity with the pancreas. It was first described in 1729 by Jean Schultz and later studied in 1970 by Potet and Duclert [1].

Majority of patients are asymptomatic. The reported incidence of ectopic pancreas in autopsy studies was 1 to 14%; it is encountered in one out of 500 abdominal surgeries [2], [3]. This uncommon submucosal lesion is mostly found in the upper gastrointestinal tract, such as the duodenum (35%), stomach (30%), and jejunum (15%); however, it can also be found in extra-abdominal locations such as the esophagus [4]. Endocrine or exocrine pancreatic tissue or a combination of both may be seen.

Malignant transformation of the aberrant pancreas is very rare. Its incidence is about 0.4% among all cases [5].

Herein, we discuss patient outcomes with a literature review of the pathology, prognosis, and management of ectopic pancreatic adenocarcinoma.

This work has been reported in line with the SCARE 2020 criteria [6].

## 2 Presentation of case

A 69-year-old man was rushed to the emergency department with feculent vomiting lasting two days. Past medical history included appendicular peritonitis, coronary bypass surgery (a couple of months before), hypertension, chronic renal failure, hypercholesterolemia, and insulin-dependent diabetes.

Vomiting occurred day and night without abdominal pain, with several months of eructation and burping; surprisingly, appetite was preserved.

Vital signs were normal; the patient's abdomen was soft and painless.

Blood analysis showed impaired renal function with creatinine of 1.8 mg/dL and C-reactive protein of 19.1 mg/L.

Due to chronic renal failure, a non-contrast abdominal CT was performed, which showed a distended stomach without any duodenal or intestinal abnormality. However, an infrarenal abdominal aortic aneurysm with a diameter > 3 cm was discovered.

The patient was hospitalized to complete investigations. He was placed on nil-per-os with parenteral nutrition, rehydration, high-dose proton pump inhibitors (PPI), and a nasogastric tube.

Upper gastrointestinal series showed severe pyloric stenosis with absent gastric emptying. We therefore decided to perform gastroscopy with biopsies. The pyloric stenosis was widened by passage of the endoscope. No lesion or ulcer was visualized. Biopsies were performed and analyzed at our hospital's laboratory; well-differentiated duodenal adenocarcinoma was considered. To complete the assessment, we decided to carry out an echo-endoscopy, and the biopsy highly indicated an antro-pyloric adenocarcinoma. At this stage, new biopsies were performed and analyzed in an external laboratory that suspected infiltration of the gastric wall by a large intestinal adenocarcinoma.

The tumor markers, carcinoembryonic antigen (CEA) and carbohydrate antigen 19–9 (CA 19.9) were within normal range.

**Fig. 1:**
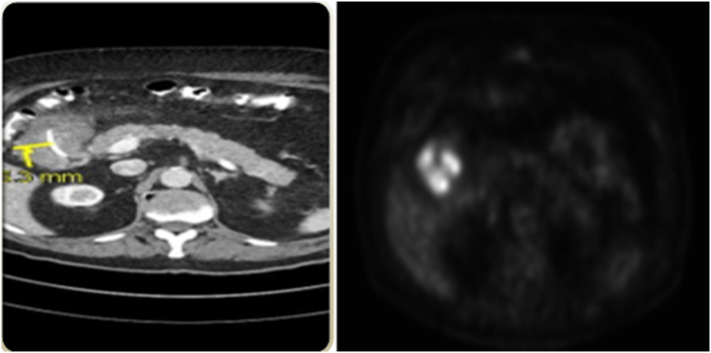
Radiologic view of the tumor a) Enhanced abdominal CT-scan: suspicious abdominal mass in the pyloric region b) Positron Emission Tomography – Computed Tomography: Hyper metabolic abdominal mass in the pyloric region, highly suspicious of neoplasia.

A PET-CT performed to exclude any metastatic lesion showed a very suspicious abdominal mass in the pyloric region (Fig. 1), without any other lesion. This indicated either a primitive cancer or a gastro intestinal stromal tumor (GIST). No abdominal or thoracic lymphadenopathy was seen.

Following this assessment, the case was discussed in a multidisciplinary meeting and surgical exploration was thus decided upon.

Intraoperatively, exploration of the abdominal cavity revealed no ascites, suspicious liver lesion, peritoneal carcinosis, invasion of the root of the mesentery, or suspicious adenopathy. However, we were immediately struck by the adhesions (which could be explained by the history of appendicular peritonitis) present at the level of the vesicular hilum, with palpation of a mass at this level. Due to the numerous adhesions, we decided to perform a partial gastrectomy and duodenectomy of D1 to resect and analyze the lesion described in the preoperative workup, and also facilitate visualization and subsequent curage of the celiac trunk and hepatic pedicle. This specimen was sent for extemporaneous analysis.

**Fig. 2:**
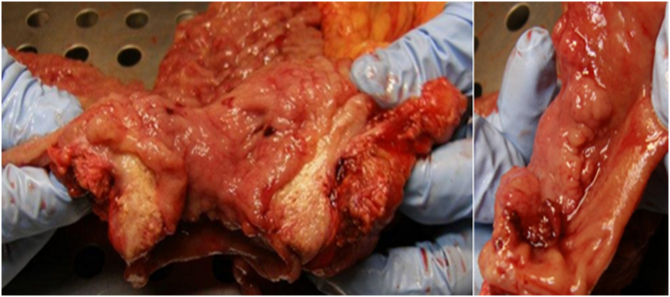
Macroscopic view of the tumor Resected piece: pyloric tumor 4 × 2 × 2 cm; mainly sub-mucous and has a whitish appearance.

However, we finally opted for a subtotal gastrectomy with a D2 lymphadenectomy without splenectomy following the extemporaneous results that described a probable gastric neoplasia (Fig. 2). A second extemporaneous analysis was performed to ensure healthy margins; this was 8 cm upstream of the lesion, allowing a subtotal gastrectomy to be performed. Also, a cholecystectomy was performed because of the fibrosis and inflammation seen opposite the vesicular hilum.

**Fig. 3:**
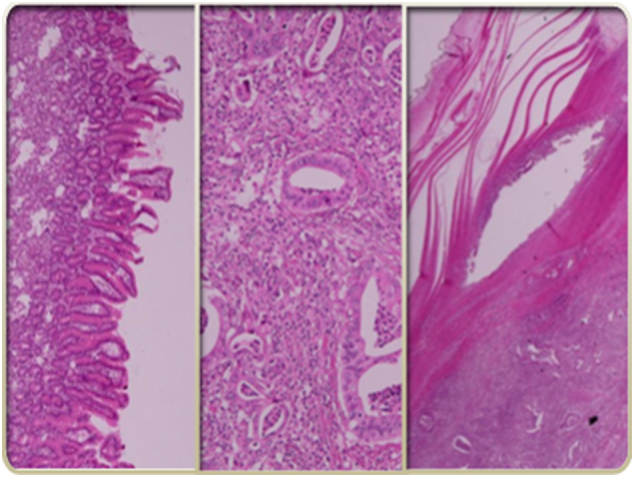
Microscopic view of the tumor Well differentiated adenocarcinoma (G1) with a “pancreatic” phenotype in hematoxylin and eosin.

The final histopathological analysis described an adenocarcinoma arising in an ectopic pancreas within the stomach wall (Fig. 3). Resection margins were free. Nine out of 18 lymph nodes were positive at analysis. The cancer was classified as pT2N3Mx. Thereafter, he underwent chemotherapy postoperatively with Gemcitabine for six months.

Twenty-two months postoperatively, recurrence occurred in form of pulmonary metastases that were suspected on CT scan and confirmed by biopsy after endobronchial ultrasound. A new chemotherapy cycle was commenced with Gemcitabine and Abraxane (Protein-bound Paclitaxel). Four year later, the lung nodules were stable. Abraxane was stopped due to stable lesions and poor patient tolerance.

## 3 Discussion

Ectopic or aberrant pancreas was first described in 1729 by Jean Schultz [7]. Aberrant pancreas can occur at any location in the abdominal cavity; however, it is usually found in the upper gastrointestinal tract. Its incidence is very low. Ectopic pancreas is usually asymptomatic but can be affected by diseases of the pancreas itself. Malignant transformation is very rare but often occurs as an adenocarcinoma. Only few cases have been reported in the literature.

Patients diagnosed with pancreatic rest are mostly asymptomatic; however, they can present with abdominal pain, bloating, gastrointestinal bleeding, intestinal obstruction or even pancreatitis.

Differential diagnosis is to be made between a gastric adenocarcinoma (because of its location) and a GIST (because the lesion is submucosal) [8]. A perioperative extemporaneous exam is necessary to exclude these diagnoses.

While ectopic pancreas may be detected with an enhanced CT scan, if a submucosal lesion is noted on upper endoscopy, an endoscopic ultrasound with biopsy is strongly recommended for further evaluation as small lesions can pass unnoticed on the CT scan [9]. Also, in 50% of cases, the cytologic examination is inconclusive; final diagnosis is reached by the histology. Heinrich in 1909 described heterotopic pancreatic types from type I to III that were modified later by Fuentes in 1973. The first type is the most common and comprises all elements of the normal pancreas, including the acini, ducts, and islet cells. The second type is dominated by acini and ducts, without islet cells, while the third type is dominated by ducts [10].

Guillou et al. [11] described three criteria that confirm an ectopic pancreatic origin of an adenocarcinoma and exclude a gastric origin. First, the tumor should be located in an ectopic pancreatic tissue or be in contact with it. Secondly, it is necessary to see a histological transition zone between pancreatic structures and the carcinoma. Finally, non-neoplastic pancreatic tissue must contain at least fully developed acini and/or islets of Langerhans. These three criteria were met in our case.

The prognosis of ectopic pancreatic adenocarcinoma is generally favorable; as seen in cases described in the literature, it seemed to show a slightly higher survival rate than orthotopic pancreatic adenocarcinoma. This could be explained by the fact that adenocarcinoma of an aberrant pancreas is more quickly symptomatic, especially when the lesion is greater than 1.5 cm [12]. Our pyloric lesion measured 4x2x2 cm which could explain the symptoms.

The management strategy of pancreatic rest should be guided by symptomatology and suspicion of malignancy. While asymptomatic lesions can be followed up, even though some authors recommend resection [13], symptomatic or malignant lesions should be resected [14]

Endoscopic resection of submucosal lesions has risk of perforation and bleeding, and should be carefully performed only when the mass is accessible [15]. In other cases or in case of a malignant tumor, surgery is indicated. In our patient, the malignant nature of the lesion and symptoms required a surgical approach. After acquiring results of the frozen section, we decided to perform a subtotal gastrectomy given the suspicion of a gastric cancer. The entire stomach could not be removed due to the presence of many adhesions following his appendicular peritonitis; however, the resection margins made us totally comfortable.

In ectopic pancreata, it would seem that limited resection with healthy margins is sufficient; however, no recommendations could be established due to the rarity of such cases.

Given the very limited number of cases of ectopic pancreatic adenocarcinoma in the literature, it is very difficult to make recommendations regarding the benefit of adjuvant or systemic chemotherapy in these situations; however, it appears that management, not only surgical but also medical, is parallel to the management of adenocarcinoma of the pancreas [16].

In cases of resectable tumors, the first surgery seems to be the reference and is followed by chemotherapy, often with gemcitabine, in case of positive lymph node, as was the case in our patient. In case of metastatic tumors, gemcitabine remains the reference chemotherapy, based on studies which included patients with metastatic and locally advanced tumors. For benefit of neoadjuvant chemotherapy, randomized trials are underway to evaluate its value [17].

## 4 Conclusion

The incidence of ectopic pancreas is low and its diagnosis is difficult but should be considered as a differential diagnosis of upper gastrointestinal submucosal lesions.

This clinical case illustrates the difficulty of establishing the diagnosis of such lesions.

The standard treatment for ectopic pancreatic adenocarcinoma is surgery with a higher survival rate of 5 years compared to pancreas cancer. Frozen section analysis is essential for deciding extent of surgery.

## Provenance and peer review

Not commissioned, externally peer-reviewed.

## Funding

This research did not receive any specific grant from funding agencies in the public, commercial, or not-for-profit sectors.

## Ethical approval

N/A

## Consent

Written informed consent was obtained from the patient for publication of this case report and accompanying images. A copy of the written consent is available for review by the Editor-in-Chief of this journal on request.

## Research registration

N/A

## Guarantor

Mansvelt Baudouin.

## CRediT authorship contribution statement

KM: Conceived, coordinated and designed of the study. Write the manuscript. Took care of the patient.

MG: Took care of the patient in the emergency service and performed the surgery.

DT: Took care of the patient in the emergency service until before surgery.

MB: Took care of the patient in the emergency service and performed the surgery.

## Declaration of competing interest

All authors disclose no financial and personal relationships with other people or organizations that could inappropriately influence (bias) this work.

The authors declare no conflict of interest.
